# Gastrointestinal Dysfunction and Dysbiosis in Ischemic Stroke: Opportunities for Therapeutic Intervention

**DOI:** 10.3390/ph18030320

**Published:** 2025-02-25

**Authors:** Rhiannon V. Macom, Candice M. Brown

**Affiliations:** 1 Department of Microbiology, Immunology and Cell Biology, West Virginia University School of Medicine, Morgantown, WV 26506, USA; rvm0005@mix.wvu.edu; 2 Department of Neuroscience, Rockefeller Neuroscience Institute, West Virginia University School of Medicine, Morgantown, WV 26506, USA

**Keywords:** stroke, GI tract, inflammation, microbiome, dysbiosis

## Abstract

Although strokes originate in the brain, it is now widely appreciated that peripheral organ systems are also impacted by stroke. The gastrointestinal system is one peripheral organ system that is impaired during ischemic stroke. This impairment results in numerous complications, which impede post-stroke recovery. Many of the gastrointestinal mechanisms that contribute to the pathophysiology of ischemic stroke remain poorly understood. This review will highlight the molecular and cellular mechanisms underlying gastrointestinal outcomes in stroke by focusing on the complex interactions that largely occur in the small intestine. The final portion of this review will focus on therapeutic interventions that target the gut as a strategy to prevent or delay functional impairment and cognitive disability in stroke patients.

## 1. Introduction

Stroke is one of the leading causes of death and disability worldwide [1,2]. The World Health Organization reports that 15 million people worldwide suffer a stroke each year. These strokes result in the death of approximately one-third of individuals who have a stroke and permanent disability to one-third off all stroke patients. Although strokes are most common in aged individuals, prevalent risk factors, including hypertension, obesity, and tobacco use, can also contribute to more severe strokes as well as an earlier age of stroke onset [3]. This review will focus on ischemic strokes, which result from a blockage of a blood vessel in the brain and comprise approximately 85% of all strokes [1,4]. In contrast, hemorrhagic strokes occur when there is a rupture of a blood vessel that causes bleeding in the brain. The primary goal of physicians who treat stroke is to suppress the growth of the necrotic stroke core and to salvage the peri-infarct cells which comprise the penumbra. Current therapeutics aim to prevent or minimize subsequent short-term and long-term disabilities such as sensorimotor deficits and long-term cognitive impairment. Current FDA-approved therapeutic options for stroke are limited, and the two primary options are either administration of recombinant tissue plasminogen activator (rtPA) or mechanical thrombectomy. Both therapeutic options have limitations related to the timing of the initiation of the stroke, location of the stroke, and patient eligibility restrictions. These limitations leave many patients without effective therapeutic strategies to reduce the morbidity and mortality associated with strokes [5,6]. Therefore, it is essential to identify and implement therapeutic strategies that target organ systems beyond the brain.

## 2. Systemic Effects of Ischemic Stroke

Recent advances in preclinical and clinical stroke research have demonstrated that strokes result in a systemic inflammatory response that affects multiple organ systems. Although the primary effects of stroke are neurological in nature, the organ systems that can be affected continue to increase and include the lung, heart, spleen, renal system, and gastrointestinal tract [7]. Stroke is a trigger for venous thromboembolism, with pulmonary embolism happening in over 1% of patients [8]. Stroke has also been shown to be linked to cardiac dysfunction, with it being more common post-stroke, as well as the association between the insular and cardiac outcomes [9,10,11,12,13]. Renal impairment has also been noted post-stroke [14,15]. The liver and kidney are important organs that are affected during stroke. Stroke outcomes are linked to liver enzymes, with National Institutes of Health Stroke Scores (NIHSS) being worse in those with altered bilirubin levels [16,17]. While kidney disease is a risk factor for stroke, kidney disease is also more common post-stroke [18,19], highlighting the importance of understanding the communication between these organs. Stroke also affects bone and muscle density, which can have detrimental effects on daily life [20,21]. Stroke can also have larger cognitive implications such as post-stroke cognitive dementia and dysfunction in sleep [22,23,24]. The systemic effects of stroke highlight the importance of better understanding the mechanisms and long-term effects of stroke within the whole body.

Gastrointestinal (GI) tract function is significantly impacted in ischemic stroke patients. The most common GI-associated complaints following stroke are GI bleeding, constipation, and dysphagia. However, the number of patients affected by GI distress varies greatly depending on the study. First, many studies that focus on GI dysfunction in stroke are retrospective rather than prospective studies. Second, the documentation of GI distress is often overlooked due to the pressing nature of other more severe and debilitating stroke symptoms. For example, one retrospective study found 8.5% of patients suffered with gastrointestinal bleeding during admission for acute ischemic stroke and that those patients had increased risks of mortality and disability [25]. Other retrospective studies found that 48% of patients suffered from constipation [26] and 20.7% suffered with dysphagia, which was then associated with pneumonia and less favorable outcomes [27]. When bowel function was evaluated in a group of stroke patients, those with constipation had a significantly prolonged colon transit time as well as more severe swallowing issues, highlighting how important and interrelated these GI issues are in this patient population [28]. Nevertheless, the clinical community would benefit from a more complete characterization of GI-related stroke outcomes because GI-specific stroke outcomes generally worsen stroke outcomes in the brain and other organ systems. Identifying and characterizing these GI specific outcomes are essential for developing appropriate therapeutic measures.

Over the past decade, multiple studies have begun to focus on systemic inflammation during stroke, with an emphasis on the interplay between the brain and gut. The gut–brain axis (GBA) is a bidirectional communication system that integrates neuroimmune pathways in health and disease [29]. The GBA participates in the regulation of innate and adaptive immune responses through immune cell trafficking and signaling during ischemic stroke. Yet many of these molecular and cellular mechanisms are not completely understood [30]. The remainder of this review will focus on the cellular mechanisms underlying GI tract dysfunction in ischemic stroke and evaluate potential therapies to prevent or suppress GI impairment, thereby decreasing disruption of the GBA in stroke.

## 3. Gastrointestinal Tract Alterations in Ischemic Stroke

### 3.1. Intestinal Barrier Function

**Figure 1:**
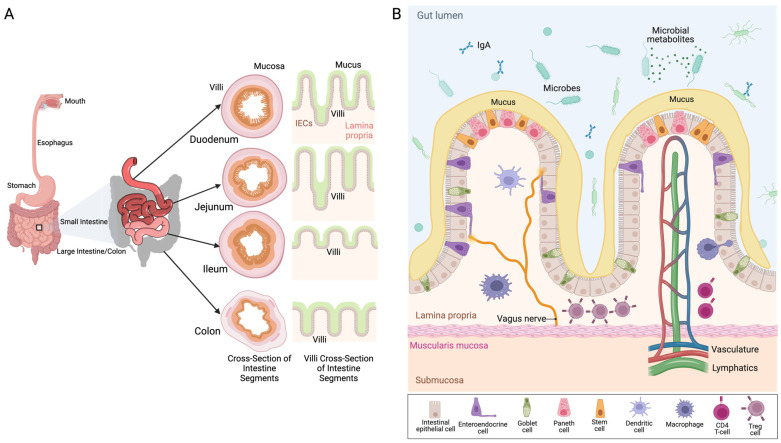
Overview of gastrointestinal tract anatomy and immune cells. (**A**) The gastrointestinal tract spans from the mouth to the large intestine, or colon. There are three distinct regions of small intestine, i.e., duodenum, jejunum, and ileum. The small intestine is followed by colon. Note that all sections of the small and large intestine that have distinct cross-sectional and longitudinal morphology. This morphology contributes to distinct roles in immune homeostasis in health and human disease. (**B**) A representative diagram of the complex interactions between immune cells, vasculature, and lymphatics within the gut lumen of the small intestine are illustrated. Together, these immune cells regulate gut immunity, bacterial translocation, and maintain barrier integrity. A legend for each cell type is depicted at the bottom of the figure. Figure generated in https://BioRender.com.

The GI tract is a complex organ system with unique physiology and immune cell populations that are critical for maintaining gut–brain axis function during healthy states and supporting a recovery to homeostasis in disease states. These features are described in Figure 1. While the GI tract plays roles in nutrient digestion, when it comes to its role in disease states, it is important to understand the intestinal barrier [31,32]. The primary function of the intestinal barrier is to aid in the maintenance of gut homeostasis by modulating transport to and from the gut lumen. Since the functions of the intestinal barrier are well described in many recent reviews [33,34,35], this review will focus on the intestinal barrier functions that are compromised in ischemic stroke. The various layers of defense within the intestinal barrier are important in regulating various functions. The mucus layer limits the exposure of the epithelial cells to the gut microbiota [36,37]. The outer mucus layer interacts with the microbiome, which is known to be altered during stroke, as well as the production of antimicrobial proteins such as immunoglobulin A [38]. Mucin is an important building block of the mucus layer that is also known to be altered in inflammation. The intestinal epithelial barrier is also a well-characterized portion of the intestinal barrier that may be altered in stroke. Intestinal epithelial cells (IECs) are important in maintaining the barrier through feedback from the gut microbiome and immune cell signaling [38]. The lamina propria is encased by the IECs and is important in regulating communication with the innate and adaptive immune cells that reside there, such as dendritic cells, T cells, B cells, and macrophages [37]. IECs include specialized cell types such as enterocytes, Paneth cells, and goblet cells. These cells are important in their ability to maintain barrier integrity and homeostasis, partly through the transport pathways. These transport pathways along with the tight junction proteins control entry and exit into the intestine, which is key in regulating inflammation [37].

Due to the complexity of the intestinal barrier, any type of barrier dysfunction and/or loss of function can be highly detrimental to intestinal homeostasis. Any imbalance of interactions within the barrier and in response to the external environment can have negative consequences. This imbalance results in a dysregulated immune response at the barrier and alters the transport of microbes, which leads to a cycle of chronic increased inflammation and, therefore, a higher risk for inflammatory disorders [37]. Intestinal barrier dysfunction and inflammation can occur when a single layer or cell type is disrupted. For example, diseases such as irritable bowel disease and celiac disease have been linked to intestinal barrier dysfunction [38]. Most importantly, loss of intestinal barrier function increases intestinal inflammation, which, in turn, can spread and accelerate acute systemic inflammation or exacerbate chronic inflammation. The knowledge of the systemic inflammatory mechanisms that link stroke and intestinal barrier dysfunction is currently limited, and more preclinical and clinical studies are needed.

### 3.2. Intestinal Motility

GI motility refers to alterations in digestion from the ingestion of nutrients via the mouth as they traverse the stomach, small intestine, and large intestine. Disrupted GI motility, or dysmotility, is the result of changes in the contractile properties of the smooth muscle cells and the enteric nervous system [34,39]. The clinical manifestations of dysmotility typically include dysphagia, dyspepsia, constipation, and incontinence [34]. These outcomes typically result in delayed gastric emptying, feeding intolerance, and intestinal obstructions [40]. Although dysmotility is well established in disorders such as irritable bowel disease and gastroparesis, the mechanistic links between altered GI motility and stroke outcomes are less clear.

One of the reasons for the lack of mechanistic studies that focus on GI dysmotility in ischemic stroke is that GI motility is typically analyzed as a secondary rather than primary outcome. Ye et al. [41] utilized a photothrombotic stroke model in eight-week-old male C57Bl/6 mice and employed the impelling ratio as a measure of motility. The results of this study showed a significant decrease in the impelling ratio at 24 h post-stroke; however, at seven days post-stroke, no differences were observed between stroke and sham mice. This result suggests that the alterations in GI motility may occur at acute timepoints and then return to normal levels over time. Conversely, Xu et al. [42] focused on ghrelin, a hormone involved in satiety, and quantified differences in both motility and ghrelin levels after MCAO in male Wistar rats. Although the authors did not evaluate changes in motility from mice with strokes compared to sham controls, quantification of intestinal motility in mice with stroke uncovered a significant negative correlation between serum ghrelin levels and the intestinal impelling force [42].

In stroke patients, alterations in motility, such as post-stroke gastroparesis, are associated with the overgrowth of intestinal bacteria, which, in turn, may contribute to infection [43]. Despite the prevalence of post-stroke infections, there are relatively few clinical studies that have investigated changes in post-stroke intestinal motility. The dearth of clinical studies is due to the limited number of methods that can effectively evaluate motility in the clinical setting as well as the expense associated with performing these methods [34,40]. For example, there are no established guidelines on how to diagnose a patient with dysmotility or the criteria to classify patients who require further treatment [40]. Recently, Muhle et al. [44] completed a retrospective single-center investigation that focused on patients in the neurological ICU that had been on mechanical ventilation. The major findings of this relatively small study identified an association between delayed gastric emptying, or decreased motility, and dysphagia severity in stroke patients. These findings are of particular interest because dysphagia is a side effect in many acute stroke patients [44]. Similarly, the PREDICT 1 clinical trial assessed the metabolic response of individuals who consumed standard meals by adding blue dye to the patient’s food as a novel measurement of motility [45]. Patients consumed muffins containing blue food dye, and the investigators measured the time from consumption until blue dye was visible in the patient’s stool. The use of blue dye provided an inexpensive method to measure motility. Coupling the use of the blue dye along with microbiome analysis also allowed investigators to identify a link between the gut microbiota composition and intestinal transit time, reinforcing the idea that GI dysfunction typically affects multiple aspects of homeostasis.

### 3.3. Intestinal Permeability

The “leaky gut” phenomenon, which is a broad way to describe alterations in GI permeability, has been linked to various diseases that are either directly or indirectly linked to the GI tract such as celiac disease, asthma, and diabetes [46]. Recent studies of the GBA have established a mechanistic role for intestinal permeability and the regulation of stroke outcomes. Intestinal permeability describes the ability to control material passing from within the gut, through the mucus layer, and on to rest of the body. There is always some permeability to allow for necessary passage; however, intestinal permeability typically increases in numerous disease states. Increased permeability results in the unregulated movement of small molecules or bacteria into and out of the gut, a process which can be detrimental. On a physiological level, intestinal permeability is a complex process that is regulated by various transport processes. Alterations in paracellular permeability occur when tight junction, adherens junction, or cytoskeletal proteins lose stability. In contrast, alterations in transcellular permeability can occur when transporter levels are disrupted and lead to shifts in permeability [46,47].

Intestinal permeability is an endpoint in many studies that have focused on the GBA in ischemic stroke. One limitation is that changes in paracellular transport are much easier to analyze than changes in transcellular transport; therefore, most stroke studies have focused on permeability changes in paracellular transport within the gut [47]. In rodent models, the most widely employed method to quantify gut permeability is the use of fluorescently labeled size-graded dextrans. The timing of dextran administration and size of the dextrans vary across studies. To quantify intestinal permeability, dextrans are typically gavaged into the stomach, given time to circulate out of the gut into the bloodstream, and then quantified in blood collected at intervals or at the termination of the study. El-Hakim et al. [48] utilized the MCAO model in male and female Sprague-Dawley rats between 5 and 7 months old. Their results showed a significant increase in the concentration of 10 kD FITC-dextran detected in the blood at 90 min post-stroke in females and at 30 min post-stroke in males. Administration of the larger 70 kD dextran molecule also showed increased permeability between 30 and 90 min post-stroke in male mice but not in female mice. These results demonstrate that permeability changes occur relatively quickly during the acute phase post-stroke. Furthermore, the increased permeability to 10 kD and 70 kD dextrans demonstrates a sex difference in post-stroke intestinal permeability [48]. Crapser et al. [49] evaluated young and aged C57Bl/6 mice after MCAO and found that permeability was increased in both groups to a small 376 Da molecule at 72 h post-stroke. Post-stroke permeability of aged mice with a larger 4 kD dextran showed a significant increase in intestinal permeability compared to shams [49]. Similarly, Ye et al. [41] evaluated mice at both one and seven days post-stroke and observed significantly increased permeability at one day post-stoke that was not sustained at seven days post-stroke. In contrast, Oyama et al. [50] saw no differences in intestinal permeability at three days post-stroke. These three studies emphasize the importance of timing during the assessment of changes in intestinal permeability post-stroke. The use of antibiotics, which is common in stroke patients [51,52], can also impact permeability. A study by Liu et al. [53] evaluated the use of antibiotic treatment by treating one group of mice with antibiotics for four weeks prior to stroke. Their results showed that treatment with antibiotics led to a significant decrease in permeability when compared to mice treated with a vehicle control.

Another indirect method to assess changes in permeability and concomitant alterations in GI morphology is to evaluate the histological differences in tissue sections. El-Hakim et al. [48] employed hemotoxylin and eosin (H&E) staining of cryosectioned ileum tissue from male rats subjected to MCAO and identified a significantly lower villi/crypt ratio post-stroke compared to female rats. A reduction in the villi/crypt ratio is an indicator of compromised intestinal function. Additionally, Periodic acid Schiff (PAS) staining and immunohistochemistry for ZO-1 also showed a loss of barrier integrity post-stroke. Stanley et al. [54] also performed PAS staining and ZO-1 immunohistochemistry in mice. Their results showed that sham mice had increased ZO-1 immunostaining, which is indicative of tight junction integrity and associated with improved barrier function. They also found that mice with strokes had increased numbers of goblet cells, which is indicative of increased mucin production associated with improved barrier function. Liu et al. [55] observed similar results with sham mice having an intact epithelium, while mice with strokes showed evidence of necrosis and shedding of villi post-stroke. Overall, intestinal histology is very beneficial for understanding the morphological changes in permeability that are not easily evaluated clinically. However, many papers that report changes in intestinal histology and/or show histological images do not attempt to quantify the extent or severity of any reported changes. Efforts to quantify intestinal pathological changes through more rigorous methods of quantification would substantially benefit the stroke research field.

Taken together, the general consensus across numerous studies is that intestinal permeability is increased post-stroke. Yet, there are many variables, such as timing of onset of permeability and the external variables, that affect permeability and confound the interpretation of results in some studies. Future efforts should focus on the evaluation of cellular and molecular mechanisms that govern intestinal permeability in stroke as well as another neurological disorders.

## 4. Gut Microbiota and Dysbiosis

Current published studies on the role of the GBA in ischemic stroke have primarily focused on the contributions of the gut microbiota to stroke outcomes. In general, gut dysbiosis is viewed as a cause or consequence of stroke and is associated with worse post-stroke outcomes. Since there are numerous excellent published reviews on the regulation of the GBA in ischemic stroke [29,56,57,58], this section will provide a brief overview of common post-stroke alterations in the gut microbiota. First, it is important to appreciate the challenges associated with studying the gut microbiota and the subsequent interpretation of results. The unique nature of an individual’s microbiome, whether human or animal, can play a large role in the outcome of gut microbiome studies. Notably, in clinical studies, it is difficult to control lifestyle characteristics such as diet, exercise, and housing conditions, which are baseline factors that contribute to higher inherent variability in disease populations. Preclinical studies also have challenges. Although the use of germ-free and/or specific pathogen free mice is useful in controlling large sources of variability in animal studies, variability across housing conditions in animal vivaria has led to inconsistent conclusions in numerous gut microbiome publications.

### 4.1. Gut Microbiome

A systematic review by Lee et al. [58] evaluated 19 studies, including clinical and preclinical, on the gut microbiome in stroke. The general conclusions were that factors such as aging and inflammation can contribute to stroke and that an abundance of Firmicutes and Bacteroidetes phyla was most highly associated with stroke outcomes. In general, a greater abundance of Firmicutes is associated with negative outcomes, while a greater abundance of Bacteroides is associated with positive outcomes [58]. A separate study [59] utilizing a pig model reported significant alterations in alpha diversity metrics at one day post-stroke followed by minimal alterations in alpha diversity metrics at three days post-stroke. Alpha diversity metrics assess differences within subjects, while beta diversity metrics assess differences between subjects. In contrast, the results from a clinical study showed that a decrease in alpha diversity within the gut microbiome correlated with three-month unfavorable outcomes in patients [60]. Despite these noted shifts in alpha diversity and reported alterations in common phyla, there are numerous other risk factors such as age and hemorrhagic transformation that contribute to gut microbiome disruption in ischemic stroke. Spychala et al. [61] utilized young and aged male C57Bl/6 mice and concluded that the gut microbiome of young mice post-stroke was highly similar to the gut microbiome of naive, aged male mice, highlighting the effects of age on the microbiome. Hemorrhagic transformation is another factor that has been shown to alter the gut microbiome [62]. This complication is the result of peripheral blood extravasation into the brain parenchyma following an ischemic stroke [63].

### 4.2. Short-Chain Fatty Acids

Alterations in short-chain fatty acid (SCFA) levels in feces or plasma, SCFA production, or SCFA metabolism have been investigated in the etiology of numerous CNS disorders, including stroke. SCFAs are metabolites that are produced in the colon by bacterial fermentation [64]. The three most common and well-studied SCFAs are butyric acid, propionic acid, and acetic acid. While the specific mechanisms by which SCFAs alter stroke outcomes are not well understood, there is a general consensus that SCFA levels decrease post-stroke, and this decrease is thought to negatively impact post-stroke outcomes [65]. Tan et al. [66] evaluated clinical samples from 140 patients with acute ischemic stroke and 90 healthy controls. The authors observed an overall decrease in SCFA producing bacteria as well as blood SCFA levels in stroke patients. This study also evaluated microbiome composition and found that SCFA levels were negatively correlated with the Firmicutes/Bacteroides ratio. This is consistent with the general interpretation of the Firmicutes/Bacteroides ratio, wherein higher ratios are associated with gut dysbiosis. Lastly, the decrease in SCFAs was also a strong predictor of poor functional outcomes in stroke patients. A preclinical report by El Hakim et al. [48] employed a rat MCAO model and did not observe any post-stroke changes in fecal SCFA levels; however, the investigators identified an important sex difference, with higher baseline SCFA levels in males compared to females. Other studies have shown that mice with an aged gut microbiota, whether due to the age of the mouse or due to fecal transplantation from an aged mouse to a younger mouse, all show diminished levels of SCFAs [61]. Furthermore, supplementation of a depleted microbiome with a fecal transplant high in SCFA was also shown to improve stroke outcomes [67]. SCFAs have been shown to aid in the repair of the intestinal barrier [53]. Supplementation with SCFA in a mouse model decreased behavioral deficits and improved neuronal circuit function post-stroke [68]. In summary, these studies demonstrate that SCFAs can modulate the composition of the gut microbiota and, by extension, immune function during stroke.

### 4.3. Trimethylamine N-Oxide (TMAO)

TMAO, similar to SCFAs, is a metabolite of intestinal microbes. TMAO is formed via the digestion of various dietary compounds, and while it is said to be correlated with disease states, including stroke and cardiac disease, some of the roles are still controversial [69]. TMAO was seen to be associated with various non-communicable diseases through a meta-analysis, including cardiovascular disease, stroke, kidney disease, and neurological diseases [70]. Other meta-analyses of research have directly correlated TMAO levels with cardiac risk [71] and, interestingly, shown that there is a positive dose-dependent relationship between circulating TMAO concentration and stroke [72]. The PEGASUS-TMI trial took plasma from patients with prior myocardial infarction and showed that higher TMAO was associated with cardiovascular death and stroke [73]. Zhu et al. utilized a mouse model and fecal matter transplant from human subjects with low or high TMAO. This study was able to show that the TMAO levels were a transmissible trait and affected stroke severity [74]. The emerging literature on TMAO continues to implicate this in stroke severity; however, more studies are still needed to evaluate the mechanisms of these alterations by TMAO as well as potential mechanisms to manipulate TMAO levels.

### 4.4. Bacterial Translocation and Infection

A major challenge in stroke recovery and significant contributor to post-stroke disability is post-stroke infection. Urinary tract infections and pneumonia are among the largest causes of post-stroke infections. In a meta-analysis of over 13,700 stroke patients, Westendorp et al. showed that 10% of patients suffered at least one infection post-stroke [52]. The current and most accepted theory to explain the origin of post-stroke infections is the bacteria that normally reside within the lumen of the GI tract, considered to be part of the normal microbiota [54], exit the lumen as part of early stroke injury, enter the bloodstream, and migrate in the bloodstream to distal organs [75]. Bacterial translocation is defined as the movement of bacteria from one location to another and most commonly refers to the movement of commensal bacteria within the GI tract into the bloodstream and beyond to other organs, such as the mesenteric lymph node (MLN) and spleen [76]. O’Boyle et al. evaluated laparotomy patients and observed bacterial translocation in 15% of patients. The authors found that the majority of septic events post-surgery were due to the translocation of normal gut flora [77]. Crapser et al. [49] observed that both bacterial translocation and organ colonization were increased three days post-stroke in mice. Age is another important factor in bacterial translocation. The authors found that, post-stroke, *E. coli* only colonized the young mice, *Enterobacter* only colonized old mice, while *Staphylococcus* and *Enterococcus* colonized both young and old mice. In contrast, Oyama et al. [50] found no evidence of bacterial translocation at 24 h post middle cerebral artery occlusion (MCAO.) Overall, these studies demonstrate that analysis of bacterial translocation post-stroke, like other characteristics of the gut microbiota, can also be challenging and that further studies are needed to understand the relationship between bacterial translocation and post-stroke infection.

In summary, the gut microbiota and its related topics such as SCFA production, metabolite production, and post-stroke infection are the most extensively researched areas in studies of the GBA and ischemic stroke. Alterations in the microbiome can have both positive and negative effects on stroke outcomes, with the quantity and types of bacteria as the most important factors. However, the microbiome is highly individualized in both humans and mice, and more rigorous clinical and preclinical studies are needed to better understand the short-term and long-term contributions of the gut microbiota to ischemic stroke.

## 5. GI Immune Cell Activation and Trafficking During Stroke

Understanding immune cell activation, trafficking, and the associated inflammatory responses within the GI tract provides important insights into the contribution of the GBA in ischemic stroke. Blood–brain barrier (BBB) disruption is known to be a major factor in stroke, and the opening of the BBB is an important component of the ischemic cascade. The discussion of the brain immune response in stroke is left incomplete without acknowledging that the brain is an immune-privileged site, which means that immune trafficking is strictly controlled. Immune-privileged sites are separated from the circulation by complex barriers, which are believed to consist of endothelial and epithelial barriers and can also be associated with inflammation. Immune cell trafficking is an important part of the immune response and, therefore, an important aspect of understanding the immune response to stroke. The gut is home to the largest immune cell population, and communication through the GBA leads to the question of how and why immune cells that reside in the gut traffic to the brain post-stroke. Current studies demonstrate that stroke leads to a response from both innate and adaptive immunity [78,79]. This section will review the contributions of immune cell modulators and immune cells that govern GI function and dysfunction during ischemic stroke.

### 5.1. Immune Cell Activation

Immune cell activation and inflammation typically coincide in CNS disorders such as stroke. Inflammasomes are innate immune system receptors that can initiate immune functions and are involved in numerous diseases [80]. In a mouse model of stroke injury, Kerr et al. [81] found that inflammasome activation was increased up to seven days post-stroke, both within the brain and the intestine. These results further establish the importance of the GBA in ischemic stroke. In another study, Yuan et al. [82] investigated the levels of pro-inflammatory mediators in the brain post-stroke. They observed an increase in interleukin 1-beta (IL-1b), tumor necrosis factor-alpha (TNFα), toll-like receptor 4 (TLR4), and CCL2 in the brain, and both TNFα and TLR4 in the gut. Interestingly, they also observed an increase in interleukin-17 (IL-17a), a cytokine involved in host defense and adaptive immunity. IL-17 is a pro-inflammatory cytokine produced by T-helper (CD4) cells that has been shown to be detrimental to ischemic injury as part of the post-stroke immune response. Zeng et al. [83] observed that aged mice had an increase in systemic IL-17 levels post-stroke and speculated that this increase was likely due to gut dysbiosis. Similarly, Benakis et al. [84] also reported an increase in IL-17 associated with ischemic injury, presumably through the translocation of IL-17 producing T cells that translocate from the gut. 

In contrast, Liu et al. [53] also assessed cytokine gene expression in the gut post-stroke and reported an increase in TLR4 as well as interleukin-6 (IL-6), NFκB, and TLR2, further showing the inflammatory response in the gut following stroke. There was also an increase in the anti-inflammatory cytokine IL-10, which, along with IL-17 due to its production by gamma delta (γδ) T cells, may play an emerging role in the intestinal immune response during stroke [53]. Interestingly, Benakis et al. [84] reported that mice deficient in IL-10 had a significant lack of neuroprotection compared to wild-type controls, which supports that IL-10 is an important cytokine in the post-stroke response.

### 5.2. Specialized T-Cell Responses: Tregs and γδ T Cells

T cells are an important part of the adaptive immune response. While it is known that T cells are part of the post-stroke immune response in both the brain and the gut, the contributions of specific T-cell subsets within these organs as well as the trafficking between these two organs are less clear. It is important to first understand the roles Tregs play in the intestine under normal conditions. Tregs have important roles in intestinal tolerance, induction, and host defense due to their role in controlling the immune response; however, as resident Tregs adapt to the intestinal environment, they also have non-immune functions, such as promoting local tissue repair and the integrity of the epithelial barrier [85]. A similar theme of many gut interactions is the gut microbiome, which can also be an important component of Tregs. Subsets of Tregs in the intestine have developed receptors that are able to recognize antigens derived from the gut microbiome, meaning Tregs are another important aspect of maintaining the microbiome and homeostasis [86].

γδ T cells are a subset of T cells that are currently thought to have a pro-inflammatory role in stroke [87]. Within the intestine, γδ T cells are located on the surface of the intestine mucosa and engage in crosstalk with microorganisms. Commensal bacteria play a role in the activation and migration of these cells in their role of monitoring for pathogens, and within the intestine they have a role in accelerating pathogen clearance and limiting the opportunistic invasion of commensal bacteria [88]. Benakis et al. [84] authored a seminal study on intestinal immune cell trafficking. This study utilized mice with intestinal dysbiosis. At 16 h post-stroke, the authors quantified a small number of γδ T cells in the ischemic brain, with a concomitant increase in γδ T cells observed in the meninges that were increased post-stroke [84]. To do this, the authors utilized a KikGR33 mouse model to track T cells migrating from the intestine to the brain. The identification of T cells in the meninges post-stroke provided confirmation that immune cells originating in the gut do travel from the gut to the brain post-stroke [84].

Similarly to the Benakis study, Brea et al. [89] conducted another elegant study on immune cell trafficking from the intestine to the CNS. CAG/KikGR mice were used to study immune cell trafficking from the gut to the brain. In this mouse model, immune cells originating in the intestine can be photoconverted as needed and then fluorescently tracked to distal organs. Initial studies in naive mice showed immune cell trafficking from the intestine into lymph nodes, spleen, bone marrow, and brain. Further studies in mice with strokes observed increased migration of immune cells from the small intestine to peripheral organs at three days post-stroke, with a significant decline in immune cell trafficking at 14 days post-stroke. At three days post-stroke, they observed increased immune cell migration from the intestine to the brain and meninges, with a similar decline observed peripherally at 14 days post-stroke. Notably, high levels of γδ T cells were also observed in the spleen at 14 days post-stroke [89]. Although immune cell trafficking to the brain in ischemic stroke remains an important research topic, these studies clearly established that the gut immune cell population traffic to other organs during ischemic stroke.

## 6. Gut-Targeted Therapeutic Interventions in Stroke

Current stroke therapeutics focus heavily on restoring blood flow to the brain and salvaging cells in the penumbra. The goal is to treat the stroke as soon as possible to save neurons; however, little thought is put into the secondary and systemic effects of stroke. With a large population of stroke patients experiencing GI dysfunction, it is important to consider what potential therapeutics may alleviate these issues or influence the GBA, leading to a better quality of life. GI-targeted therapeutics for stroke have the potential to improve GI dysfunction and dysbiosis as well as long-term neurological outcomes related to sensorimotor function, cognitive impairment, and overall quality of life. Currently, GI-targeted stroke therapeutics target the modulation of the gut microbiota through direct manipulation (FMTs, probiotics), indirect manipulation (SCFA, alternate drugs), or modulation of the immune response and gut barrier (intestinal epithelial stem cell transplants). These therapeutic strategies are summarized in Table 1 and described in further detail below.

### 6.1. Diet

**Figure 2:**
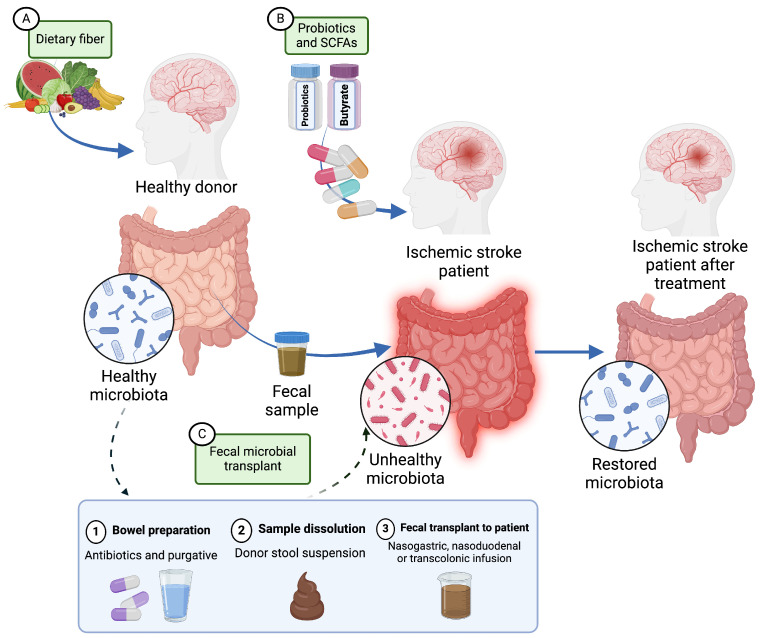
Summary of clinically tested therapeutic approaches to treat ischemic stroke by modulation of the gut microbiome. (**A**) Addition of dietary fiber is a preventative strategy that can increase beneficial gut bacteria. (**B**) Probiotics and short chain fatty acid (SCFA) supplementation can be administered post-stroke to modulate gut dysbiosis. (**C**) Utilization of fecal microbiome transplants (FMT) can be administered from a healthy donor to a stroke patient to ameliorate gut dysbiosis and improve overall stroke outcomes. The steps of the FMT donation procedure are also summarized. Figure generated in https://BioRender.com.

Several studies have shown a clear association between diet and stroke risk and/or stroke outcome. O’Donnell et al. observed that diet was a modifiable risk factor for stroke by comparing populations in 32 countries across six continents, representing high- and low-middle-income countries [90]. Another study by Peng et al. [91] found that a pro-inflammatory diet was associated with increased stroke risk. This study found that a high dietary inflammation index (DII), indicative of a pro-inflammatory diet, was associated with an increased carotid atherosclerotic plaque vulnerability in patients with ischemic stroke. Vulnerable plaques are an unstable collection of macrophages and lipids that increase the risk of stroke. In contrast, some dietary practices are associated with a decreased stroke risk. Helte et al. [92] evaluated women in the Swedish Mammogram Cohort and reported that higher levels of calcium in drinking water led to a decreased risk for both ischemic and hemorrhagic strokes. Other studies have found that coffee and tea consumption have been shown to decrease stroke risk [93]. In addition, calorie restriction in preclinical animal models of stroke has been shown to have beneficial effects post-stroke. Although dietary alterations have been proposed as a potential therapeutic intervention to ameliorate stroke-associated GI dysfunction, implementing dietary interventions in stroke patients is largely impractical. Taken together, a more practical strategy would be to implement dietary interventions in individuals at high risk of stroke as a therapeutic option to minimize the incidence and severity of future strokes (Figure 2). Alternatively, therapeutics that incorporate SCFAs may be a more promising therapeutic avenue. These treatments are designed to enrich beneficial SCFA populations, which, in turn, leads to improved stroke outcomes. Administration of SCFAs in drinking water [68], as well as treatment with SCFAs, such as b-hydroxybutyrate [94], indole-3-propionic acid [95], and sodium butyrate [96], has led to improved post-stroke outcomes in animal models.

### 6.2. Antibiotics

Antibiotics have also been tested as potential gut therapeutics with equivocal results. Although Liu et al. [53] showed that antibiotic treatment in an animal model led to a decrease in infarct volume as well as brain and systemic cytokines, current clinical research does not support antibiotics as a therapeutic against GI-associated dysfunction and infection in stroke. While current clinical research does support the use of antibiotics as a therapeutic to limit overall infections in stroke patients, studies that employed a targeted antibiotic regimen to decrease urinary tract infections in stroke patients did not observe decreased mortality, decreased incidence of pneumonia, or improved neurological outcomes in this patient population [97,98,99]. However, further evaluation of the aforementioned studies suggests that a subpopulation of stroke patients have the potential to benefit from targeted antibiotic treatment [100].

Table 1 describes potential stroke therapeutics targeting the gut–brain axis that have been evaluated in clinical studies. Studies are separated by therapeutic class.

**Table 1:** Preclinical studies for stroke therapeutics that target the gut–brain axis.

Potential Therapeutic	Reference	Animal Model	Stroke Model	Experimental Paradigm	Results
**Probiotics**					
Probiotics	[101]	Male C57Bl6 mice, 25–30 g	Transient middle cerebral artery occlusion (tMCAO)	Vehicle or probiotics were given for 14 days pre-stroke	Decrease in infarct size, malondialdehyde, and tumor necrosis factor-alpha
Probiotics	[102]	Male Sprague-Dawley rats, 250–300 g	MCAO	IV injections of inactivated *Lactobacillus* (ILA) 2 hours pre-stroke	ILA administration led to better neurological scores and decreased infarct volume
Probiotics	[103]	Male ICR mice, 6 weeks old	Cerebral I/R via bilateral common carotid artery occlusion	200 mL of *C. butyricum* daily for 2 weeks pre stroke	*C. butyricum* administration improved neurological deficits, increased butyrate in the brain, and decreased caspase-3 expression
Lactulose	[104]	Male C57BL/6 mice, 6–8 weeks old	Photothrombotic stroke (PTS)	Beginning 24-hours post-stroke mice received control or lactulose for 2 weeks via oral gavage	Decrease in stroke neurological severity scores, foot vault scores, lesion volume, inflammatory factor expression, and immune cell response.
**Fecal Microbial Transplant (FMT)**					
FMT and dietary modulation	[84]	Male mice, 10 weeks old	tMCAO	Calorie restriction and fecal microbiome transplant	Caloric restriction led to better long-term rehabilitation; FMT from calorie restricted mice also led to better long-term rehabilitation
FMT	[87]	Male C57BL/6 mice, 18–20 months old	tMCAO	Fecal matter transplant was administered from healthy young or aged mice	Mice who received FMT replicated healthy donor microbiome profiles; young FMT improved behavioral recovery, altered intestinal and brain immune cells, and increased SCFAs.
FMT	[51]	Male Sprague-Dawley rats, 9 weeks old	tMCAO	Rats were grouped into control, antibiotic, or FMT groups	FMT restored SCFA decrease and improved gut dysbiosis
**Short Chain Fatty Acid-Based**					
Beta-Hydroxybutyrate (BHB)	[94]	Male Sprague-Dawley rats, 10–12 weeks old	MCAO	BHB administered into lateral ventricle during MCAO	Moderate doses of BHB reduced neurological scores and decreased infarct volume
Short chain fatty acids	[68]	Male C57BL/6 mice, 6–8 weeks old	PTS, distal MCAO, filamentous MCAO	SCFA administered in water for 4 weeks	SCFA administration improved recovery of limb motor function, altered contralesional cortex connectivity, and impacted microglial activation
Indole-3-propionic acid	[95]	Male C57BL/6 mice, 22 g	MCAO	Intragastric administration of indole-3-propionic acid (IPA) post-stroke until euthanasia	Treatment with IPA led to a restoration of control microbiome, repaired intestinal barrier, and decrease in infarct
Sodium butyrate (NaB)	[105]	Female Sprague-Dawley rats, 9–11 months old	Endothelin-1 induced MCAO	Rats were treated with NaB at 6- and 30-hours post-stroke	Treatment with NaB led to a decrease in infarct volume, sensory motor impairment, and inflammatory cytokine levels
**Others**					
Intestinal epithelial stem cell (IESC) transplant	[106]	Male and female Sprague-Dawley rats, 5–7 months and 12 months old	MCAO	IESCs from young rats were injected via tail vein injection at 4-, 24-, and 48-hours post-stroke into aged rats	IESC transplants restored intestinal dysmorphology and permeability, decreased LPS and IL17A, and improved behavioral outcomes
Antibiotics	[53]	Male Wistar rats, 4 weeks old	Endothelin-1 induced MCAO	Rats were treated with broad spectrum antibiotics for 4 weeks pre and 2 weeks post stroke	Antibiotics led to a decrease in systemic and brain cytokines and infarct size and improved behavioral outcomes
Atorvastatin	[86]	Male C57BL/6 mice, 12 weeks of age	Permanent middle cerebral artery occlusion (pMCAO)	Atorvastatin or control administered by oral gavage	Improved neurological function, reduced infarct volume, mediates inflammation, increases fecal butyrate levels, recovers intestinal integrity
Dietary modification	[96]	Male C57BL/6 mice, 8 weeks old	Intraluminal MCAO	Mice were fed a normal diet (ND) or protein-reduced (PRD) for 56-days post-stroke	Post-stroke PRD led to a decrease in infarct size and better neurological and motor recovery compared to ND. ND mice had reduced fecal microbiome at 7-days post-stoke while PRD had restored bacterial diversity.

### 6.3. Probiotics and Prebiotics

Although research on GI-targeted therapeutics is limited, most therapeutic strategies have focused on ways to manipulate the gut microbiota [107]. Probiotics, prebiotics, and symbiotics are potential therapeutics that can be administered in pill form (Figure 2). Probiotics are live bacteria, prebiotics are fuel for existing microorganisms, and symbiotics are a mixture of the two. Akhoundzadeh et al. [108] administered probiotics to mice who were subjected to MCAO. While treatment with probiotics did not affect neurological function, they found a significant decrease in infarct volume along with a decrease in malondialdehyde and TNFα in ischemic tissues. While clinical studies on probiotics administration during stroke are limited, a recent meta-analysis by Zhong et al. [109] evaluated patients who received enteral nutrition (EN) alone compared to those who received EN and probiotics following stroke. This meta-analysis revealed that while NIH stroke scores (NIHSS) did not change during the period of observation, patients who received probiotics had shorter hospital stays. Further analysis of clinical studies showed that C-reactive protein (CRP) levels, a marker of systemic inflammation, were not altered, while other cytokines, such as TNFα, IL-6, and IL-10, were all decreased. Importantly, intestinal-specific outcomes including intestinal stress and infection were also decreased with probiotics and EN. Similarly, Chen et al. [110] reported that EN with probiotics led to a decrease in infection and dysbiosis compared to those with EN only.

### 6.4. Fecal Microbiome Transplants (FMTs) 

Fecal microbiome transplants (FMTs) have also shown promise in modulating the gut microbiota and improving stroke outcomes (Figure 2). The bacterial and SCFA composition of transplanted fecal matter is critical and remains a focal point of many FMT investigations. Huang et al. [111] examined the impact of caloric restriction and FMT in stoke outcomes. The authors found that caloric restriction in mice led to improved stroke outcomes, specifically by shifting the gut microbiota toward an increase in *Bifidobacterium,* bacteria associated with health benefits. FMT from these calorie-restricted mice also led to a translation of these positive outcomes. The effects of stroke as well as the composition of the microbiota are known to change with age. Lee et al. [112] showed that FMTs from young mice to aged mice improved the gut microbiota composition and, subsequently, SCFA composition, which led to overall improved stroke outcomes. Similarly, Chen et al. [67] demonstrated that administration of FMTs rich in SCFA that were supplemented with butyric acid also led to improved stroke outcomes. Thus, FMTs could be a viable therapeutic strategy to improve GI dysfunction by restoring the gut microbiota in stroke patients.

### 6.5. Emerging Therapeutic Strategies

Aside from studies on the regulation of the gut microbiota, FMTs, and administration of SCFAs, investigations are limited on alternative therapeutic strategies. Each alternative therapeutic will be discussed briefly. First, current strategies to modulate GI dysfunction in stroke also repurpose drugs used in other disorders. Other therapeutics have been administered in stroke, such as lactulose [82], a laxative used to treat stroke-associated constipation, and atorvastatin [113], a member of the statin class of cholesterol-lowering drugs, and have been shown to have positive benefits in stroke. While this approach can be effective, it does not directly address the therapeutic need.

Second, stem cells have long been investigated for the treatment of difficult diseases such as stroke. Various animal studies have shown that neural stem cells that are transplanted are able to survive and could be utilized as a potential therapeutic [114,115]. Undifferentiated mouse embryonic stem cells [116] and induced pluripotent stem cell-derived neural stem cells [117] were shown to improve post-stroke outcomes. A recent study utilized intestinal epithelial stem cell (IESC) transplants to evaluate alterations in post-stroke outcomes. This is particularly interesting because it is the first study to evaluate gut stem cells for brain repair. The key finding of this study was that IESCs from healthy young donors given to aged stroke animals led to repair of the gut and attenuation of stroke-induced cognitive impairment [106]. Lastly, IL-17 has known links to gut dysfunction that may support its status as a promising therapeutic target. This has led to speculation that the use of anti-IL-17 antibodies may be a promising drug class to ameliorate GI dysfunction in stroke patients, since anti-IL-17 antibodies are under investigation as a potentially promising therapeutic target for other inflammatory diseases [118,119,120].

## 7. Conclusions

This review highlights the importance of maintaining gut homeostasis as well as the consequences of gut dysbiosis. Understanding the mechanisms of gut dysfunction in stroke as well as the mechanisms of potential therapeutics along with the potential systemic interactions will lead to more effective stroke therapies. Many of the studies that are currently published show a lack of attention to sex and age. These variables are important to consider due to the sex differences in stroke as well as the consideration of stroke being an age-related disease.

Although our understanding of intestinal mechanisms in ischemic stroke has expanded over the past decade, outstanding questions exist that impede our ability to develop therapeutic strategies to address this critical health issue. These include the following: (1) What are the molecular and cellular mechanisms underlying GI dysfunction in stroke? (2) How do intestinal glial cells such as astrocytes and microglia contribute to GI dysfunction and GBA modulation during stroke? (3) Which neuronal circuits contribute most significantly to the disruption of the GBA during ischemic stroke? (4) How do gut-targeted stroke therapies alter systemic stroke outcomes? Thus, future studies and research tools are desperately needed to identify interventions that shift the GI tract and gut microbiota toward outcomes leading to improved bacterial composition, diminished intestinal permeability, and controlled immune responses. Overall, this research field would also benefit from a more comprehensive understanding of how the gut microbiota influences brain and systemic immune responses and inflammation post-stroke. This new knowledge, in turn, can be used to develop better therapeutics, which specifically target GI dysfunction, to reduce morbidity and mortality in stroke patients.
